# Supplementary material for: A Multi-Tissue Gene Expression Atlas of Water Buffalo (Bubalus bubalis) Reveals Transcriptome Conservation between Buffalo and Cattle
Source: Genes (Basel). 2023 Apr 10;14(4):890. doi: 10.3390/genes14040890 (PMC10137413; doi:10.3390/genes14040890)
Supplement: Supplementary file 1 [file genes-14-00890-s001.zip › genes-2328423-supplementary.pdf]

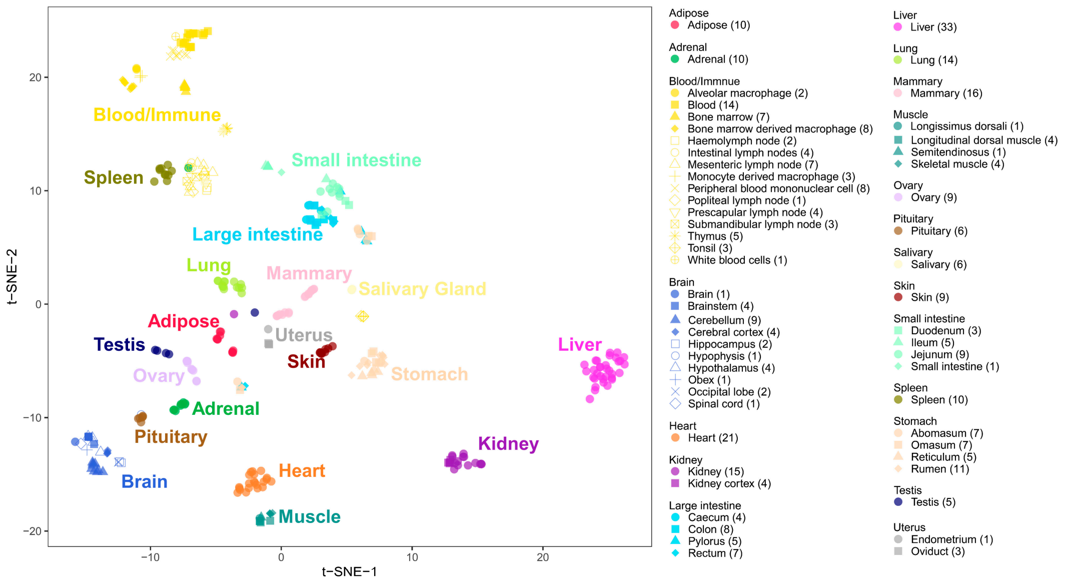

Figure S1. Plot of t-SNE of samples based on gene expression. Each dot represents a sample, colored by 20 tissue classes, shaped by 57 tissue details. Parenthetical numerals represent the sample size for each tissue.

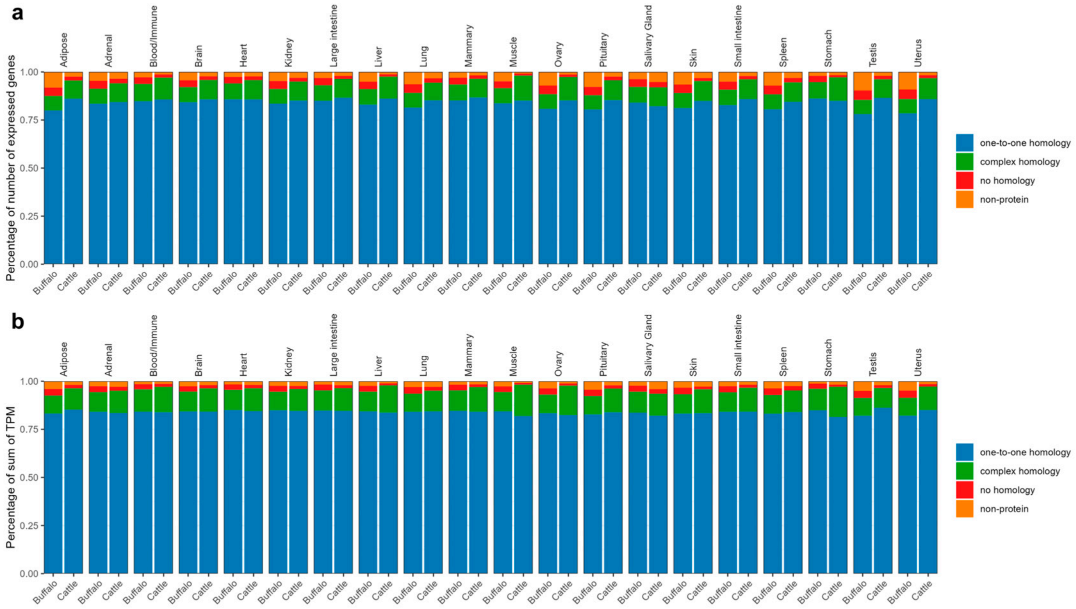

Figure S2. The percentage of expressed genes and summed expression in the four categories. (**a**) The proportion of expressed genes in each category to the total number of expressed genes (TMP > 0.1). (**b**) The proportion of summed gene expression ($\log_2$(TMP)) in these categories to the total expression. Genes are classified into four categories: one-to-one homology (blue), complex homology (green, including one-to-many, many-to-one, or many-to-many), no homology (red), and non-protein (orange).

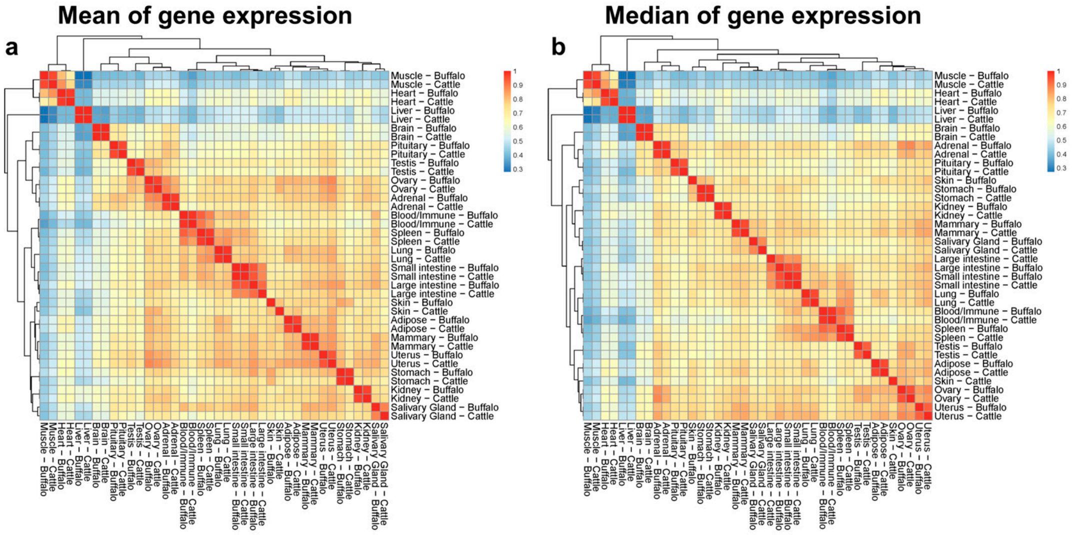

Figure S3. Hierarchical clustering of tissues in buffalo and cattle. Heatmap of hierarchical clustering of tissues based on Pearson's correlation of mean expression (**a**) and median expression (**b**) of Orthologous genes.

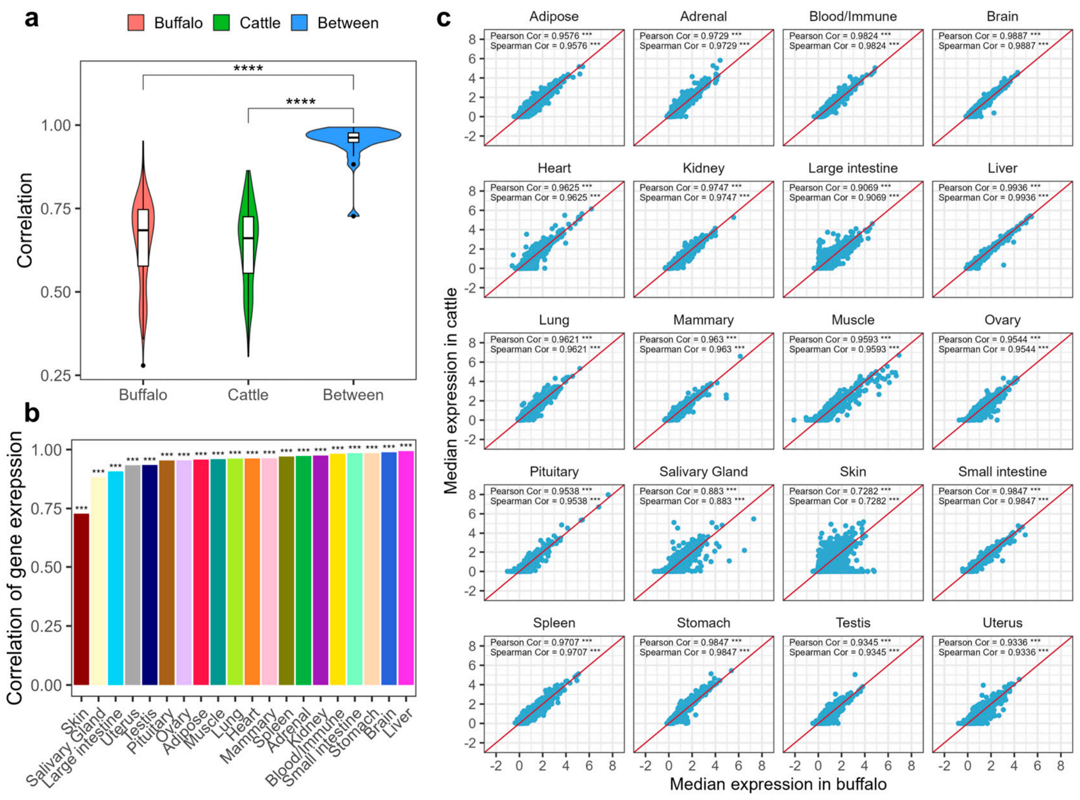

Figure S4. Comparison of gene expression between buffalo and cattle tissues. (**a**) Violin plot compares three groups of correlations, (1) between tissues within buffalo (red), (2) between tissues within cattle (green), and (3) within tissues between species (blue). "****" represents the adjusted *p*-value (FDR, one-side Student's *t*-test) less than 0.0001. (**b**) Bar-plot shows the correlations of gene expression in each of 20 tissues between buffalo and cattle. "****" represents the adjusted P-value (FDR) less than 0.0001. (**c**) Scatter-plot shows the median expression of each tissue in buffalo and cattle, with the red line representing a slope of 1. The Pearson and Spearman correlation coefficients of gene expression for each tissue between buffalo and cattle, along with their corresponding levels of significance, are indicated above each graph. "***" represents the adjusted *p*-value (FDR) less than 0.0001.

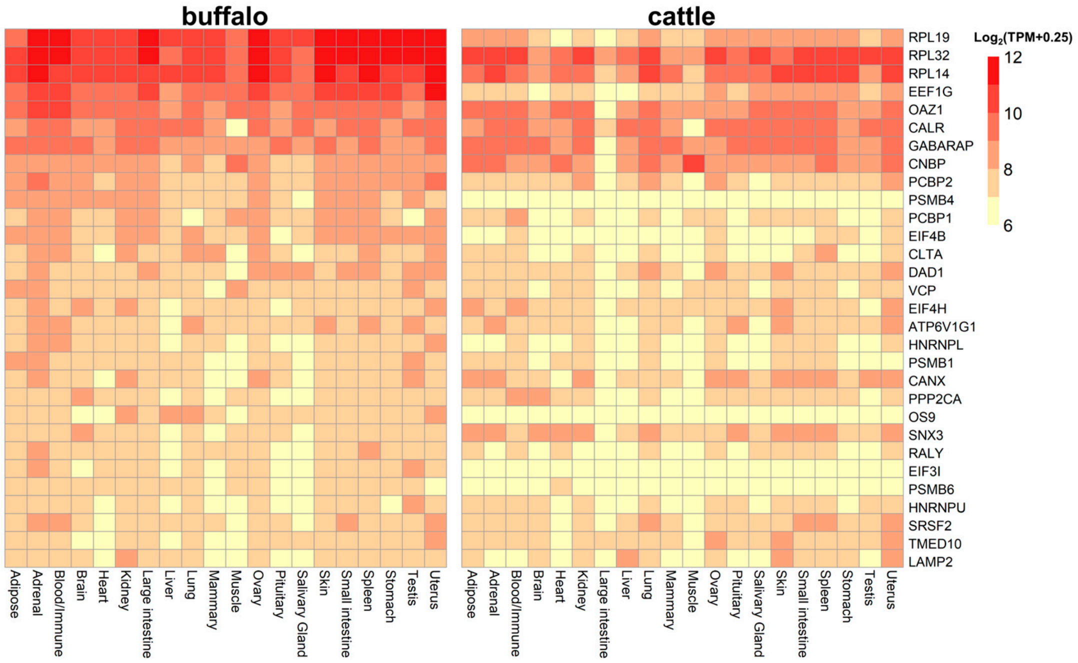

Figure S5. Heatmap of gene expression of top 30 highly expressed HKGs in buffalo and cattle. The top 30 highly expressed HKGs were derived from genes exhibiting consistently high expression in both buffalo and cattle, and ranked based on their median expression across different tissues in buffalo.
